# High dose cefuroxime causing retinal toxicity in a patient undergoing trabeculectomy

**DOI:** 10.1016/j.ajoc.2022.101343

**Published:** 2022-02-02

**Authors:** Jae Yee Ku, Shiao Wei Wong, Laura R. Steeples, Claire Delaney, Neil R.A. Parry, Cecilia Fenerty

**Affiliations:** a Department of Eye and Vision Science, Institute of Life Course and Medical Sciences, University of Liverpool, Liverpool, L7 8TX, United Kingdom; b Manchester Royal Eye Hospital, Manchester University NHS Foundation Trust, Manchester Academic Health Science Centre, Manchester, M13 9WL, United Kingdom; c Faculty of Biology, Medicine and Health, University of Manchester, United Kingdom

**Keywords:** Cefuroxime toxicity, Uveitis, Cataract, Trabeculectomy, Imaging, Retinal toxicity

## Abstract

**Purpose:**

To present a case of severe retinal toxicity secondary to high dose intracameral cefuroxime administered during trabeculectomy glaucoma surgery. We describe the clinical features and management, and describe serial multimodal imaging and electrophysiological findings. Intracameral cefuroxime (ICC) and subconjunctival cefuroxime (SCC) are routinely administered during ocular surgeries to prevent postoperative endophthalmitis. Cefuroxime toxicity with both standard (1mg/0.1mL) and high doses of ICC (2–100mg) and SCC (31.25mg) have been reported. To the best of our knowledge, this is the first report of cefuroxime retinal toxicity in trabeculectomy surgery, which is of particular significance because of the possible differences in pharmacokinetics within the eye.

**Observations:**

A 69-year-old male with primary open-angle glaucoma, underwent right trabeculectomy, augmented with mitomycin C (0.2mg/mL). The patient inadvertently received cefuroxime 12.5mg/0.1mL as an intracameral rather than a subconjunctival injection. Within 4 hours, the error was discovered and the patient underwent immediate anterior chamber (AC) washout. His right best-corrected visual acuity was hand movements, and he rapidly developed uveitis including AC cells and moderate vitritis and haze. Optical coherence tomography (OCT) demonstrated serous macular detachment, characteristic schisis-like changes in the outer nuclear layer and ellipsoid zone disruption. Multi-focal electroretinograms (ERG) identified deficits undetected by full-field ERG. He was successfully managed with intensive local topical corticosteroid, non-steroidal therapy and peri-ocular corticosteroid injection.

**Conclusions and importance:**

As ICC and SCC are routinely used in intra-ocular surgery to prevent endophthalmitis, ophthalmologists need to be aware of this potential complication and consider this in patients with unexplained reduced vision post-operatively. Theatre teams need to be vigilant about potential dilution and administration errors to ensure that the correct concentration and volume of cefuroxime is given via the correct route. We highlight the risks of high dose intracameral injection, including uveitis and retinal toxicity, and the utility of serial OCT, and full-field and multi-focal ERGs in this condition. We report a favourable outcome with significant and rapid improvement in retinal structure and function observed during follow-up. A literature review of the condition is presented.

## Highlights

• Consider cefuroxime toxicity in patients with reduced vision in the early post-op period.
• OCT typically shows serous macular detachment and schisis-like changes in the outer nuclear layer.
• Be vigilant about potential dilution and administration errors during ocular surgery.
• OCT and electrophysiology are useful to diagnose and monitor cefuroxime toxicity.

## 1 Introduction

Cefuroxime is a broad-spectrum cephalosporin antibiotic routinely used in ocular surgery for prophylaxis of post-operative infections. In cataract surgery, intracameral cefuroxime (ICC) became widely accepted, following a multicentre study by the European Society of Cataract and Refractive Surgeons (ESCRS) which demonstrated that ICC administration reduced the incidence of post-op endophthalmitis by five-fold.^1^ However, there have been reports of ocular toxicity with standard (1mg/0.1mL)^2^, ^3^, ^4^, ^5^, ^6^, ^7^, ^8^, ^9^, ^10^ and high doses (2–100mg) of ICC,^11^, ^12^, ^13^, ^14^, ^15^, ^16^, ^17^, ^18^, ^19^, ^20^ including retinal toxicity, corneal toxicity and uveitis, with a risk of permanent visual loss from retinal structural damage.^7^^,^^14^^,^^21^

In trabeculectomy surgery, subconjunctival cefuroxime (SCC) rather than ICC is usually given for antibiotics prophylaxis because the procedure itself increases filtration of aqueous humour from the anterior chamber (AC) and consequently reduces aqueous humour cefuroxime concentration. A higher dose and different concentrations of cefuroxime are administered with adjustment for diffusion into the eye via adjacent ocular structures to prevent endophthalmitis.^22^ We describe the presentation and management of severe cefuroxime toxicity, including retinal structural and functional changes, in a patient who inadvertently received high dose ICC meant for subconjunctival administration during trabeculectomy surgery. To our knowledge, this is the first report of cefuroxime toxicity in trabeculectomy surgery as previous cases were in cataract surgery.

## 2 Case report

A 69-year-old male with bilateral primary open-angle glaucoma, underwent right eye trabeculectomy, augmented with mitomycin C 0.2mg/mL, under subtenons anaesthetic. His past ocular history includes bilateral cataract surgery, high myopia, congenital red-green colour blindness and a previous penetrating eye injury in his left eye when he was 18-years-old. He had undergone a left eye trabeculectomy 9 years earlier and a right eye selective laser trabeculoplasty 6 years ago. He had no history of uveitis.

He had no significant past medical history. His pre-operative medications were oral acetazolamide (250mg QID) and timolol eye gel 0.1% (BD), bimatoprost 0.01% (OD), brinzolamide 1% (TID) and brimonidine 0.2% (BD) all in the right eye. Pre-operatively, the best-corrected visual acuity (BCVA) were −0.06 right and 0.62 left logarithm of the minimum angle of resolution (logMAR). His intraocular pressures (IOP) were 16 mmHg right and 6 mmHg left and his central corneal thicknesses (CCT) were 538μm right and 508μm left. His right eye examination pre-operatively was unremarkable except for myopic changes.

The patient received cefuroxime (Reig Jofre®) diluted to 125mg/mL at the end of his surgery, and approximately 12.5mg/0.1mL was given as an intracameral rather than a subconjunctival injection. The trabeculectomy procedure was uneventful. Standard subconjunctival dexamethasone (3.3mg/mL) was given. A few hours later, the intracameral antibiotic administration error was discovered upon review of his surgical records. The patient returned to the hospital for an urgent assessment. His right eye BCVA was hand movements. There was no relative afferent pupillary defect (RAPD), the cornea was clear, the IOP was 15 mmHg and a few pigment cells were noted in the AC. A well-draining bleb was noted. Fundoscopy of his right eye showed clear vitreous and a dull foveal reflex.

Within 4 hours of the trabeculectomy, the patient underwent AC washout under subtenons anaesthetic. The volume exchanged with balanced salt solution (BSS) was 22mL. Further subconjunctival dexamethasone (3.3mg/mL) was given. He was admitted and given prednisolone 1% 1 hourly day and night, chloramphenicol 0.5% (QID) and atropine 1% (OD) in the right eye. Acetazolamide was stopped post-operatively.

**Fig. 1:**
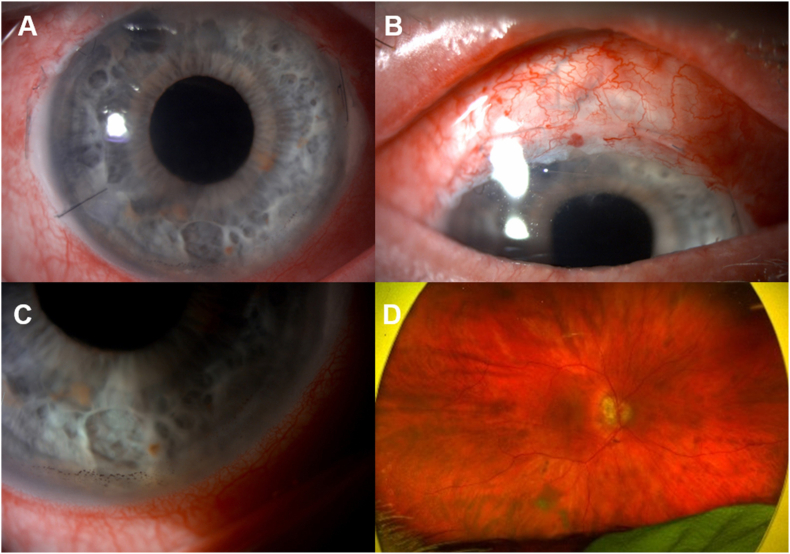
Right eye of the patient at day 1 post-op showing a clear cornea, with a peripheral iridectomy at 11 o'clock and a paracentesis wound at 8 o'clock (A) with inferior endothelial pigment (A and C); vascularised well-draining bleb (B) and Optos fundus showing a dull foveal reflex and myopic changes (D).

**Fig. 2:**
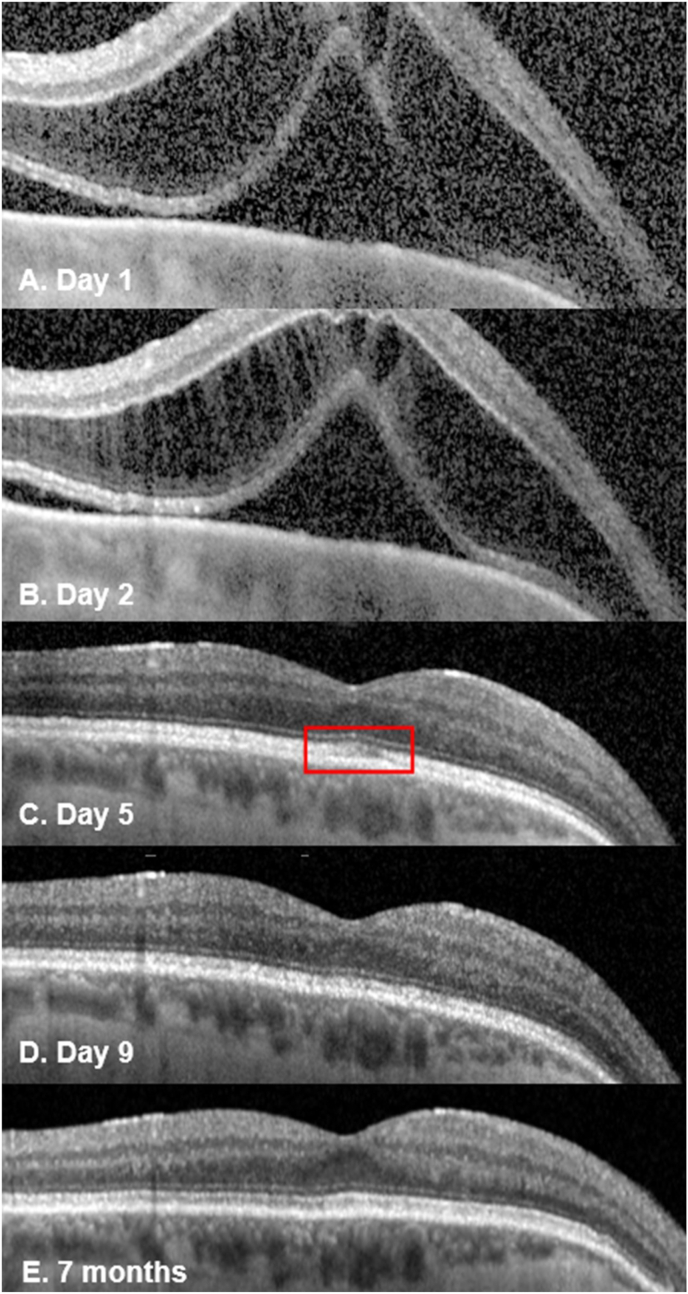
Spectral-domain OCT (Spectralis Retinal OCT; Heidelberg Engineering, Germany) of patient's right eye showing serous macular detachment (SMD) with schisis-like disruption in the outer nuclear layer (ONL) with a central subfield thickness (CST) of 794μm day 1 post-op (Fig. 2A). By day 2, the subretinal fluid (SRF) had reduced and the CST decreased to 682 μm (Fig. 2B). By day 5, the schisis-like changes had resolved with slight residual disruption of the ellipsoid zone (red box; Fig. 2C) which improved further by day 9 (Fig. 2D) and resolved at the last visit at 7 months post-op (Fig. 2E). (For interpretation of the references to colour in this figure legend, the reader is referred to the Web version of this article.)

Day one post-operatively, his BCVA were 0.88 and 0.44 logMAR in his right and left eye, respectively. The cornea was clear (Fig. 1A) and the CCT was unchanged. The right eye had AC cells (+2), flare (1+) and vitritis with moderate vitreous haze (Fig. 1). Colour vision was reduced (test plate only in each eye). The IOP was 2 mmHg in the right eye and the bleb was draining well (Fig. 1B). Fundoscopy showed myopic features with a dull foveal reflex, and no choroidal folds or detachments (Fig. 1D). Optical coherence tomography (OCT) of his right eye showed a large serous macular detachment (SMD) and schisis-like changes in the outer nuclear layer (ONL) (Fig. 2A). He had further subconjunctival dexamethasone (3.3mg/mL) and continued intensive topical corticosteroid (Prednisolone 1% 1 hourly), plus nepafenac (TID) was started.

**Fig. 3:**
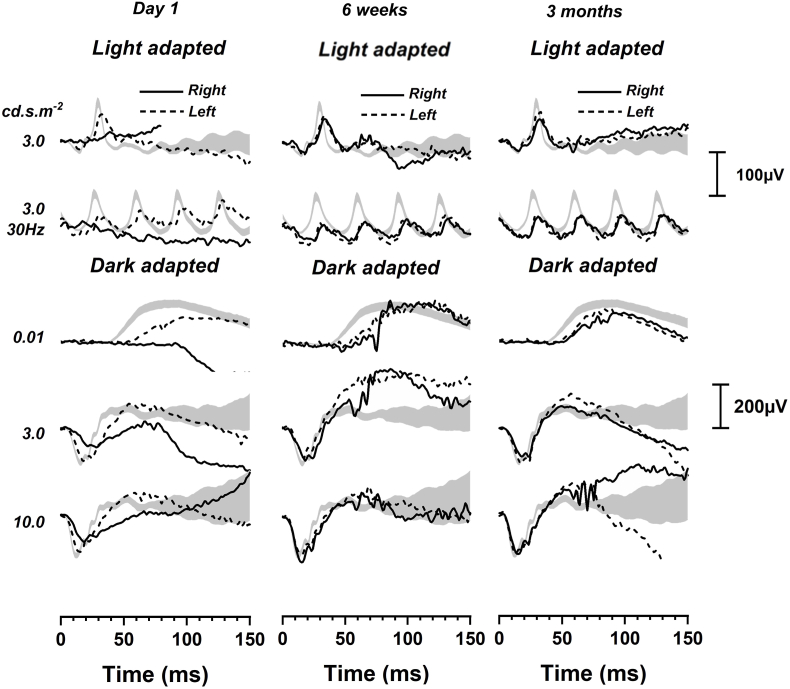
At day 1 post-op, ISCEV full-field ERG showed extinguished light adapted 3.0 single flash and 30Hz flicker responses in the right eye (solid black line) compared to the left eye (dotted black line), which was normal. The dark adapted responses were attenuated with a very low b-wave. At 6 weeks, the responses were symmetrical in both light adapted and dark adapted conditions. The right eye remained stable at 3 months. Grey areas are 95% CI from a normal database.

**Fig. 4:**
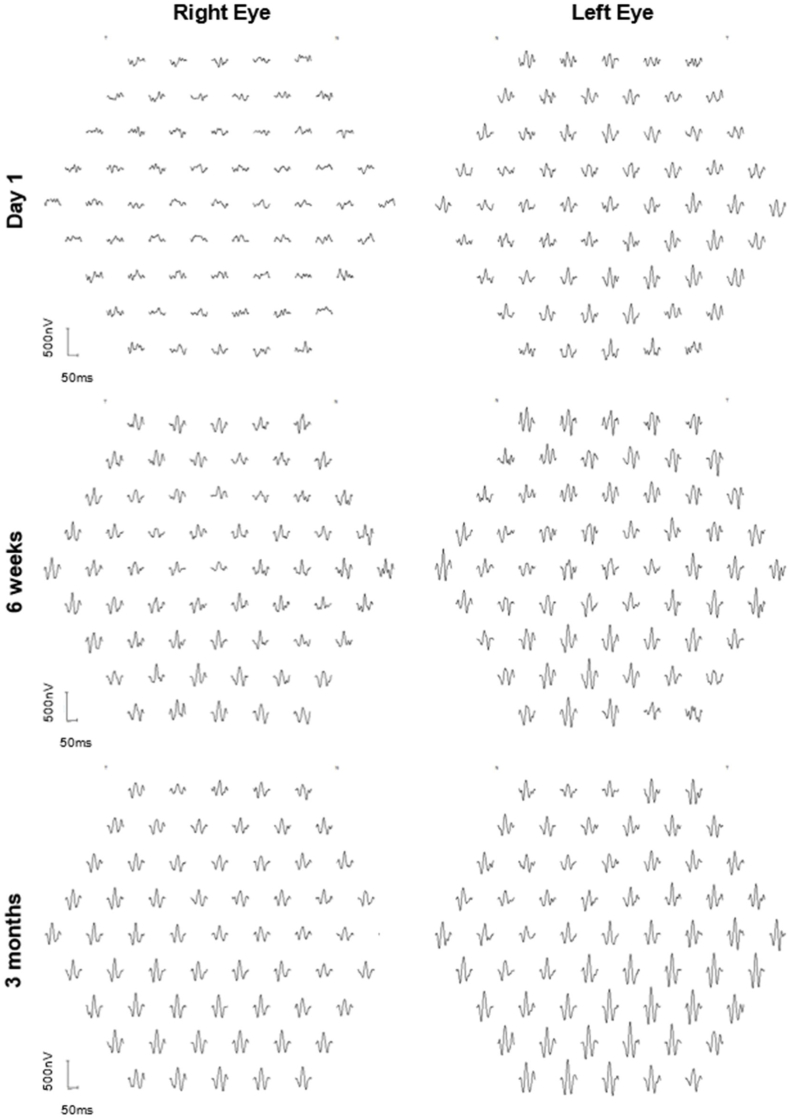
Multi-focal ERG at day 1 post-op showed reduced signals in all rings in the right eye. At 6 weeks, there were reduced signals in the central foveal region in the right eye and further improvement at 3 months. Signals were within normal limits in the left eye.

The patient underwent full-field electroretinogram (ffERG) and multi-focal electroretinogram (mfERG), which were performed according to the International Society for Clinical Electrophysiology of Vision (ISCEV) standards.^23^^,^^24^ Tests were performed using the Espion version 6.63.26, Diagnosys, Cambridge, UK. On day 1 post-op, his ffERG showed extinguished light-adapted 3.0 single flash and 30Hz flicker responses in the right eye compared to the left eye. The dark-adapted responses were attenuated with very low b-waves in DA 3.0 and DA 10.0 (Fig. 3). The left eye showed a slight delay in implicit time, which was thought to be an age-related change. mfERF showed reduced signals in all rings in the right eye while signals were within normal limits in the left eye (Fig. 4).

On day 2, his BCVA in the right eye had improved to 0.78 logMAR and the IOP was 4 mmHg. There were persistent AC cells and vitreous cells and haze. He received orbital floor depomedrone 40mg/1mL and continued topical therapy. Repeat OCT showed persistent marked structural disruption in the ONL and SMD (Fig. 2B).

**Fig. 5:**
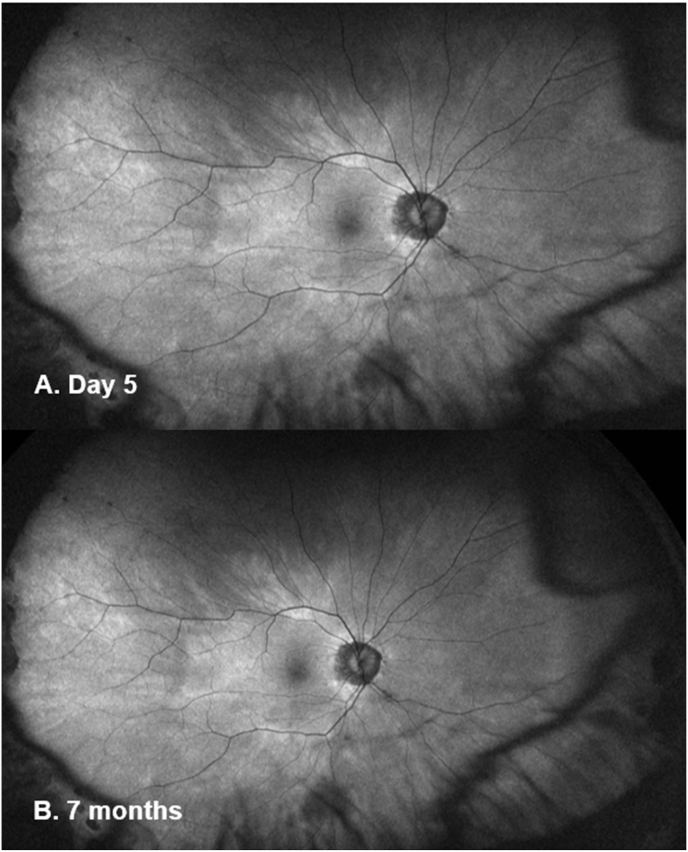
Ultra-widefield fundus autofluorescence (Optos imaging) of the patient's right eye at day 5 (A) and 7 months post-op (B) showed no macular abnormalities. Myopia related peripapillary atrophy was noted.

**Fig. 6:**
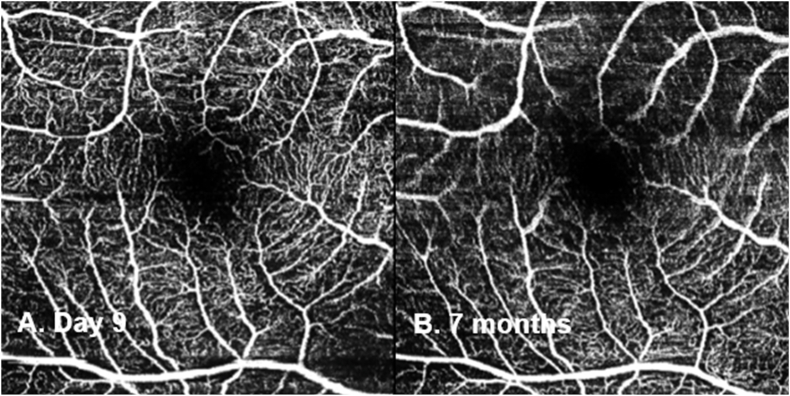
OCT angiography (Spectralis Retinal OCT; Heidelberg Engineering, Germany) of the patient's right eye at day 9 (A) and 7 months post-op (B) showed no abnormalities.

On day 5, the subretinal fluid (SRF) had resolved and a slight disruption within the ellipsoid zone (EZ) (Fig. 2C) was noted, which improved by day 9 (Fig. 2D). The intraocular inflammation was completely resolved by week 2. The central subfield thickness (CST) improved to 266μm on day 5 and remained normal during follow-up (Fig. 2E). The BCVA progressively improved to 0.12 logMAR at week 3 and 0.02 logMAR at week 6. At 6 weeks, his ffERG showed symmetrical responses in both eyes in light adapted and dark adapted conditions and remained stable at 3 months (Fig. 3). The mfERG results at 6 weeks showed reduced signals in the central foveal region in the right eye and further improvement at 3 months (Fig. 4). The endothelial cell count (ECC) were normal in both eyes (right 2640, left 2068 cells/mm^2^). No macular abnormalities were seen on fundus autofluorescence (Fig. 5) and OCT-angiography (OCTA) (Fig. 6). Topical steroid was tapered and discontinued over 8 weeks.

At the last follow-up at 7 months, the BCVA was 0.02log MAR and the IOP was 9 mmHg in his right eye. The right ffERG remained stable and mfERG remained reduced, which indicated persistent macular dysfunction.

## 3 Discussion

Since the landmark ESCRS endophthalmitis study, ICC has been widely used in cataract surgery to prevent post-operative infection.^1^^,^^25^ Standard dose ICC (1mg/0.1mL) is generally considered safe.^25^ However, ocular toxicity secondary to cefuroxime has been reported with standard^2^, ^3^, ^4^, ^5^, ^6^, ^7^, ^8^, ^9^, ^10^ and higher doses (2–100mg) of ICC.^11^, ^12^, ^13^, ^14^, ^15^, ^16^, ^17^, ^18^, ^19^, ^20^ A summary of published reports including the clinical features and management is presented in Table 1. In cataract surgery, toxicity secondary to subconjunctival cefuroxime has also been reported and is thought to be due to the passage of the drug into the eye through corneal wounds.^26^ A variety of clinical features have been reported including corneal toxicity, anterior and panuveitis, macular oedema, optic neuropathy and retinal infarction.^13^^,^^14^^,^^21^

**Table 1:** Summary of case reports of ocular side effects from intracameral cefuroxime (ICC) or subconjunctival cefuroxime (SCC).

Study	Dose (mg)	No. of eyes	Ocular side effects	Treatment	Recovery
**Current case report**	ICC 12.5	1	• AC cells, flare, dull foveal reflex, vitritis with moderate vitreous haze • OCT: SMD, schisis like changes in ONL, EZ disruption • Full-field ERG: decrease a- and b-wave amplitude • mfERG: reduced signals in all rings	• AC washout • Topical CCS • Topical NSAID • SC CCS • Orbital floor steroid	• Normal OCT within 9 days • mfERG: residual reduced signal at 7 months
**Díez-Álvarez et al.**^21^**(2021)**	ICC 12.5	8	• Corneal oedema, marked anterior segment inflammation • ECC: reduced • OCT: early macular oedema	• Periocular steroid	• Permanent disruption of EZ in 1 case • Optic neuropathy with RAPD in 1 case • ECC reduced <1000 cells/mm^2^ in 3 cases
**Chlasta-Twardzik et al.**^20^**(2020)**	ICC 1	1	• Diffuse macular oedema • OCT: SMD and macular oedema at the ONL • FFA: early phase - non-fluorescent zone next to the optic disc, surrounded by an irregularly fluorescent band; late phase - diffusing fluorescein from the periphery of the non-fluorescent zone reaching the optic disc	• Topical CCS • Topical NSAID • Oral NSAID • Oral acetazolamide • Oral Pentoxifylline	• OCT normal within 10 days with no recurrence in 24 months
**Bryan et al.**^8^**(2019)**	ICC 1	1	• Absent foveal reflection with diffuse macular oedema • OCT: SMD and macular oedema at the ONL • FFA: normal	• Topical CCS • Topical NSAID	• OCT normal within 2 weeks with no recurrence in 6 months
**Kamal-Salah et al.**^13^**(2019)**	ICC 12.5	14	• Non-infectious panuveitis, colour alteration • OCT: disruption of EZ	• Topical steroid	• Permanent disruption of EZ in 3 cases
	ICC 10	5	• OCT: SMD, IRF, disruption of EZ	• Topical steroid • Topical NSAID	• Permanent disruption of EZ in 1 case
**Andreev and Svetozarskiy**^9^**(2018)**	ICC 1	1	• OCT: SMD	• Oral acetazolamide • Subtenon CCS • IV CCS	• OCT: Residual inner retina abnormalities observed at 5 months
**Sül and Karalezli**^7^**(2018)**	ICC 1	1	• Corneal oedema, AC and vitreous inflammation, retinal haemorrhages and oedema, retinal capillary loss at 1 month • OCT: foveal thinning and outer segment atrophy • FFA: extensive vascular leakage	• IV and oral CCS	• Optic nerve atrophy with retinal neovascularization • FFA showed extensive retinal infarction at 5 months
**Zuo et al.**^6^**(2018)**	ICC 1	20	• Mild corneal oedema ± mild AC inflammation, macular oedema • OCT: SMD and macular oedema involving ONL, EZ and interdigitation zone disruption	• Topical CCS	• Cornea oedema and AC inflammation resolved after 1 week • Macular oedema and SMD resolved by 1 week in 19/20 eyes • Persistent abnormalities in subfoveal EZ and interdigitation zone after 1 week in 9 cases
**Aslankurt et al.**^18^**(2016)**	ICC 1	8	• OCT: IRF with SMD	• Topical CCS	• Recovery in 1–4 weeks
**Ricci et al.**^10^**(2016)**	ICC 1	3	• OCT: SMD and macular oedema involving ONL	• Topical CCS • Topical NSAID • Topical naphazoline	• OCT: resolution of macular oedema and SMD by 1 week • Persistent focal defect of the photoreceptor outer segment at week 1 in 2 cases
**Faure et al.**^3^**(2015)**	ICC 1	1	• Retinal pallor with diffuse retinal oedema • OCT: SMD, schisis like changes in ONL • Full-field ERG: decrease a-wave and b-wave amplitude	• Observation	• Normal OCT by 1 week • Full-field ERG normal within 2 months
**Longo et al.**^2^**(2015)**	ICC 1	5	• OCT: SMD, IRF in outer retinal layers	• Topical steroid • Topical NSAID • Oral NSAID • Oral acetazolamide	• Normal OCT within 7 days with no recurrence at 6 months
**Wong et al.**^17^**(2015)**	ICC 9	13	• Mild central corneal oedema in 2 eyes • OCT: Macular oedema in 6 eyes	• Topical CCS • Topical NSAID	• Resolution of central macular thickening within 7 days • Trace hyporeflective spaces in the OPL and subretinal space, which resolved by week 9 in 1 case
**Xiao et al**.^4^**(2015)**	ICC 1	2	• Absent foveal reflection • OCT: SMD, IRF in ONL, EZ preserved	• Topical CCS • Topical NSAID	• Normal OCT within 7 days
**Çiftçi et al.**^19^**(2014)**	ICC 50-70	4	• All patients had vitreous loss during cataract surgery • Corneal oedema, retinal haemorrhage, optic atrophy, macular pucker	• Not reported	• Optic atrophy
**Kontos et al.**^26^**(2014)**	SCC 31.25	1	• OCT: SMD, IRF in ONL • FFA: mild patchy choroidal filling	• Topical CCS • Topical NSAID • Oral NSAID	• Normal OCT within 6 days with no recurrence at 6 weeks
**Le Dû and Pierre-Kahn**^5^**(2014)**	ICC 1	6	• Corneal oedema • OCT: SMD and macular oedema in outer retinal layers	• Topical CCS • Topical NSAID • Oral acetazolamide	• Resolution of macular oedema and SMD in all cases • Functional impairment related to photoreceptor damage on OCT in late-stage in 3 cases
**Olavi**^12^**(2012)**	ICC 10-100	16	• Cloudy oedematous cornea, transient rise in IOP, pigment precipitates in the AC, loss of corneal endothelial cells, pigmentary changes on the retina, colour vision defect, defects in dark adaptation • Visual field: lowered threshold • Full-field ERG: slowing of a-wave and b-wave in scotopic rod response	• Not reported	• Corneal oedema resolved in weeks
**Delyfer et al.**^16^**(2011)**	ICC 40-50	6	• Moderate AC inflammation with fibrin, corneal oedema, increased IOP, slight vitreous haze, retinal thickening • ECC: decreased • OCT: SMD, macular oedema in ONL • FFA: Diffuse leakage • Full-field ERG: reduced scotopic b-wave amplitude	• Topical CCS • Topical NSAID • Oral Acetazolamide • Topical apraclonidine	• OCT significantly improved in 5 days, normal within 6 weeks
**Qureshi and Clark**^14^**(2011)**	ICC 62.5	1	• AC inflammation • OCT: IRF • FFA: macular infarction with macular oedema	• AC washout • IVT CCS	• Macular infarction
**Buyukyildiz et al.**^11^**(2010)**	ICC 2	2	• Trace cells in AC • OCT: SMD, IRF in ONL • FFA: window defect over the macula	• Topical steroid • Oral acetazolamide • IVT CCS	• Normal OCT within 7 days • Macular oedema recurred after 1 month and treatment reinitiated with no subsequent recurrence of macular oedema after 6 months in 1 case
**Sakarya and Sakarya**^15^**(2010)**	ICC 3	6	• Normal ocular examination, no other investigations performed	• AC washout	• No long-term adverse effect

**Abbreviations:** anterior chamber (AC), corticosteroid (CCS), electroretinogram (ERG), multi-focal ERG (mfERG), ellipsoid zone (EZ), endothelial cell count (ECC), fundus fluorescein angiogram (FFA), intracameral cefuroxime (ICC), intraocular pressure (IOP), intraretinal fluid (IRF), intravenous (IV), intravitreal (IVT), nonsteroidal anti-inflammatory (NSAID), optical coherence tomography (OCT), outer nuclear layer (ONL), outer plexiform layer (OPL), relative afferent pupillary defect (RAPD), serous macular detachment (SMD), subconjunctival (SC), subconjunctival cefuroxime (SCC).

The characteristic retinal OCT findings of cefuroxime toxicity include large SMD and schisis-like change in the ONL. Disruption of the EZ has been reported in some cases.^6^^,^^13^^,^^21^ In most cases, the SMD and ONL disruption resolve quickly within a week (Table 1). Permanent disruption of the EZ is associated with a worse visual prognosis.^13^ The accumulation of SRF and intraretinal fluid (IRF) may indicate an impairment of the retinal pigment epithelium (RPE) pump.^6^ The mechanism of this pattern of OCT changes remains unclear but ERG findings may help us understand this further.

ERG findings from this case report and other studies suggest cefuroxime affects photoreceptors’ function.^16^ Faure et al.^3^ found a mild decrease in a-wave and b-wave amplitude that resolved after 2 months in a patient who received standard dose ICC. Delyfer et al.^16^ found decreased scotopic b-wave amplitude with no change in photopic b-wave amplitude that suggested rod photoreceptors dysfunction in patients who received high dose ICC (40–50mg).

Shahar et el^27^ compared the effects of low dose (1mg) and high dose (10mg) intravitreal (IVT) cefuroxime on retinal function in rabbit models. They found that high dose cefuroxime can cause a permanent decrease in ERG amplitudes 3 hours after injection; the b-wave was reduced to a larger extent compared to the a-wave, which is consistent with findings from this case report. Shahar et al.^27^ postulated that cefuroxime induces l-glutamate transporter 1 (GLT1) overexpression in the distal retinal neurons, which lower l-glutamate concentration in the synapses of the outer plexiform layer (OPL). This leads to depolarisation of the ON-center bipolar cells and reduced amplitude of the light-induced electrical activity of these neurons. ERG improvement started with the normalisation of the a-wave and b-wave ratios. Histology sections of the rabbits’ eyes showed loss of photoreceptor outer segments, disorganisation of the layered retinal structure and retinal thinning. There was also increased expression of the glial fibrillary acidic protein (GFAP) in Müller cells, indicating retinal stress.^27^ Faure et al.^3^ suggested that the OCT appearances of cefuroxime toxicity are similar to nicotinic acid maculopathy. This is a possibility because nicotinic acid maculopathy has been postulated to be linked to Müller cell engorgement.^28^, ^29^, ^30^ In this case report, mfERG showed residual deficits at 3 months that were not revealed by the ffERG, which normalised within 6 weeks (Fig. 3, Fig. 4). Therefore, mfERG may be useful in future cases to assess long-term retinal function.

Studies have shown that high dose cefuroxime can cause corneal toxicity.^12^^,^^21^ Olavi^12^ found oedematous corneas with loss of corneal endothelial cells in 16 eyes given between 10 to 100mg of ICC during cataract surgery. Yoeruek et al.^31^ reported dose-dependent cefuroxime toxicity in cultured human corneal endothelial cells with toxicity observed in doses above 2.75 mg/mL. In another in vitro study, Haruki et al.^32^ found corneal damage at 24 hours in cefuroxime concentrations over 0.5mg/mL but not at 6 hours even at higher concentrations. Considering that aqueous humour is replenished at the rate of 1% per minute in a normal eye, for the standard dose of 1mg of ICC, the concentration of cefuroxime in the AC would decrease to 28% (0.76mg/mL) within 1 hour.^33^ In our patient, there was no clinical evidence and no reduction in ECC during follow-up. We assume the trabeculectomy surgery increased aqueous outflow and decreased the AC concentration of cefuroxime more rapidly. Furthermore, AC washout was rapidly performed with an exchange of 22mL of BSS, a volume over 100 times of typical aqueous volume.^34^

While cefuroxime corneal toxicity seems to be dose and time-dependent,^32^ retinal toxicity appears to occur more rapidly with the effect peaking at 3 hours.^27^ In our patient, despite having a successful trabeculectomy with a good draining bleb, which would increase AC flow and an AC washout within 4 hours, he still had evidence of retinal toxicity. Sakarya and Sakarya^15^ managed high dose ICC (3mg) with immediate AC washout and they noted no long-term adverse effects on clinical examination alone. However, OCT and ERG were not performed to evaluate for any structural changes.

In cases where high dose ICC was given, most were due to dilution errors.^11^, ^12^, ^13^^,^^15^, ^16^, ^17^^,^^21^ Çiftçi et al.^19^ reported 4 cases whereby the correct concentration (1mg/0.1mL) was provided but in a larger (10mL) syringe and 50–70mg of ICC were inadvertently given in eyes that also had posterior capsule rupture (PCR) during cataract surgery. In a case similar to this one, Qureshi and Clark^14^ reported a procedural mishap whereby there was confusion with the syringe containing BSS, which was also used with a blunt cannula and the subconjunctival dose of cefuroxime was delivered intracamerally. Following this case, our hospital's local theatre policy has been reviewed to ensure that the syringe containing cefuroxime will not be used with a blunt cannula.

In cases where standard dose ICC was given, there may be an increased risk in eyes with PCR and vitreous loss during cataract surgery, which is presumed to be due to ICC diffusing into the retina more readily and therefore, surgeons may need to reduce ICC dose.^7^ Bryan et al.^8^ reported cefuroxime toxicity with a standard dose of ICC in a vitrectomised eye; the authors postulated that previous epiretinal membrane removal may have weakened the inner retinal layers making the retina more vulnerable. However, even in cases where standard dose ICC was used, there could be an undetected dilution error or the surgeon may have unknowingly injected over 0.1mL. In eyes that have shown initial susceptibility to standard dose ICC, surgeons may need to consider surgery in the second eye without ICC or a dose reduction.^9^

Ocular cefuroxime toxicity after cataract surgery has been managed with various strategies ranging from observation to topical corticosteroid and nonsteroid anti-inflammatory drugs (NSAID), oral NSAID, oral acetazolamide, periocular corticcosteroid injections and AC washout (Table 1). In this case report, urgent AC washout, intensive topical and local corticosteroid plus NSAID were successfully used. Although studies have used oral acetazolamide to treat macular oedema, this was avoided in this case where the post-operative IOP was low following successful trabeculectomy and further IOP reduction from acetazolamide would have risked severe hypotony and visual loss.

Our patient developed severe structural and functional complications from cefuroxime toxicity with very characteristic OCT changes that correlated with a marked reduction in ERG responses. Significant recovery was observed with the restoration of OCT changes and visual acuity to normal limits and the improvement in ERG function, however, some mfERG changes persisted.

## 4 Conclusions

In conclusion, we demonstrated severe ocular retinal toxicity due to high dose ICC administration. As ICC and SCC are commonly used in ophthalmology practice, ophthalmologists need to be aware of this potential complication. Worse visual prognoses may be associated with higher doses of cefuroxime, permanent disruption of the EZ and posterior capsular rupture with vitreous loss during surgery. OCT and mfERG are recommended in suspected cases for diagnosis and follow-up of retinal toxicity.

## Patient consent

The patient consented to the publication of the case in writing.

## Authorship

All authors attest they meet the current ICMJE criteria for authorship.

## Funding

Funding was obtained from the 10.13039/501100000836University of Liverpool to publish in this journal.

## Declaration of competing interest

None of the authors has any financial disclosures.
